# Test–retest reliability of the FitMáx©-questionnaire in a clinical and healthy population

**DOI:** 10.1186/s41687-023-00682-9

**Published:** 2024-01-04

**Authors:** Renske Meijer, Goof Schep, Marta Regis, Nicole E. Papen-Botterhuis, Hans H. C. M. Savelberg, Martijn van Hooff

**Affiliations:** 1 Department of Sports and Exercise, Máxima Medical Centre (Máxima MC), Veldhoven, The Netherlands; 2 Department of Mathematics and Computer Science, University of Technology, Eindhoven, The Netherlands; 3 Academy, Máxima Medical Center (Máxima MC), Veldhoven, The Netherlands; 4 Department of Nutrition and Movement Sciences, NUTRIM School of Nutrition and Translational Research in Metabolism, Faculty of Health, Medicine and Life Sciences, Maastricht University, Maastricht, The Netherlands

**Keywords:** Cardiorespiratory fitness, Patient-reported outcome, Questionnaire, Peak oxygen uptake, Cardiopulmonary exercise testing

## Abstract

**Purpose:**

The FitMáx© was developed as a questionnaire-based instrument to estimate Cardiorespiratory Fitness (CRF) expressed as oxygen uptake at peak exercise (VO_2peak_). Test–retest reliability is a clinometric measurement property, which defines stability over time if multiple measurements are performed (i.e. reliability). The present study aimed to assess the test–retest reliability of the FitMáx©-questionnaire in different patient groups.

**Patients and methods:**

A total of 127 cardiac, pulmonary and oncology patients and healthy subjects aged 19–84 years who completed the questionnaire twice within an average of 18 days were included for analysis. Participants were in a stable clinical situation (no acute disease or participating in a training program). To determine the test–retest reliability, the Intraclass Correlation Coefficient (ICC) and Standard Error of the Measurement (SEM) was calculated between the first (T_0_) and second (T_1_) administration of the questionnaires.

**Results:**

An excellent agreement was found between the FitMáx©-questionnaire scores at T_0_ and T_1_, with an ICC of 0.97 (SEM 1.91) in the total study population and an ICC ranging from 0.93 to 0.98 (SEM 1.52–2.27) in the individual patient groups.

**Conclusion:**

The FitMáx©-questionnaire proves to be reliable and stable over time to estimate CRF of patients and healthy subjects.

*Trial registration* NTR (Netherlands Trial Register), NL8846. Registered 25 August 2020, https://trialsearch.who.int/Trial2.aspx?TrialID=NL8846

**Supplementary Information:**

The online version contains supplementary material available at 10.1186/s41687-023-00682-9.

## Introduction

Cardiorespiratory fitness (CRF) is an important variable that influences several health outcomes including quality of life [1, 2]. Cardiopulmonary exercise testing (CPET) is the gold standard to objectively measure CRF expressed as the oxygen uptake at peak exercise (VO_2peak_) and is clinically used to determine the underlying cause of limitations in exercise capacity [3–5]. However, CPET is costly and labour-intensive, whereas Patient-Reported Outcome Measures (PROMs) are a simple, safe and cost-effective alternative, especially in repeated testing such as rehabilitation programs [6, 7].

Máxima Medical Centre (MMC) developed the FitMáx©-questionnaire (FitMáx), which consists of only three single-answer, multiple-choice questions [8]. The FitMáx was developed to estimate cardiorespiratory fitness expressed in VO_2peak_ based on the self-reported maximum capacity of walking, stair climbing and cycling. The FitMáx scores are combined with subject’s age, sex and Body Mass Index (BMI) to estimate VO_2peak_. A previous validation study showed a strong correlation between VO_2peak_ estimated by the FitMáx (FitMáx-VO_2peak_) and VO_2peak_ measured with CPET (CPET-VO_2peak_), r = 0.94 (0.92‒0.95), ICC = 0.93 (0.91–0.95), and Standard Error of the Estimate (SEE) of 4.14 ml/kg/min. Moreover, FitMáx performed superiorly over commonly used questionnaires such as the Veterans Specific Activity Questionnaire (VSAQ) and Duke Activity Status Index (DASI) [8–10].

The clinical usefulness and applicability of PROMs depend on several clinometric properties including validity, responsiveness and reliability [11, 12]. Reliability is defined as the extent to which test results of subjects (whose condition has not changed) are the same over time. To assess such test–retest reliability of an instrument, repeated measures are performed under the same conditions [11, 13]. In this way it is possible to quantify the proportion of total variance in repeated measurements that is due to true differences in PROMs. The measurement error describes the systematic and random error of subjects’ results that are not caused by true changes in the construct to be measured [11].

The present short report aimed to assess the test–retest reliability of the FitMáx in four different groups (healthy subjects, pulmonary, oncology, and cardiac patients) and in the total study population.

## Material and methods

### Setting

Pulmonary, oncology, and cardiac patients were recruited prospectively in MMC, Veldhoven and Eindhoven, the Netherlands. Healthy subjects were included at Ancora Health in Eindhoven, the Netherlands. The authorized Medical Research Ethics Committee of the MMC has reviewed the study protocol and concluded that the rules laid down in the Medical Research Involving Human Subjects Act (also known by its Dutch abbreviation WMO), do not apply to this study (reference number N20.086). The study was registered as NL8846 in the Netherlands Trial Register.

### Study population

Subjects were eligible for inclusion if they were aged ≥ 18 years, had a good command of the Dutch language, and if no change in CRF was expected within 31 days from enrollment date. During their visit to MMC or Ancora Health, cardiac and pulmonary patients and healthy subjects who were scheduled to perform CPET, either for medical reasons or as part of a health check, were asked to participate in a study about CRF questionnaires. The CPET protocol is extensively described in our validation study [8]. Since oncology patients do not perform CPET as part of standard care, they were included from the outpatient clinic of the sports department without performing a CPET. Oncology patients were not eligible for inclusion when they were undergoing active disease-specific treatments, potentially affecting their CRF, within the study period. Similar to our validation study, subjects were asked to complete the FitMáx, VSAQ and DASI questionnaires. The questionnaires were administered in a paper format twice to the same subject. Subjects were excluded from analysis if the FitMáx was incomplete, or if the period between T_0_ and T_1_ was > 31 days. To minimize a possible ‘subject expectancy effect’, it was explicitly not explained that this was a study to determine the test–retest reliability of these questionnaires. All participants received a second information letter and questionnaire (T_1_) two weeks after T_0_. We did not explicitly question participants about experienced change in CRF. All participants gave written informed consent to the use of their anonymized CPET and questionnaire data.

### Statistical analysis

We performed a sample size calculation with an expected ICC of 0.85, a minimum acceptable ICC of 0.60 and two measurements per individual, requiring a sample size of n = 26 per subject group to achieve a power of 80%.

Statistical analyses were performed using R, version 4.2.1 (R Foundation for Statistical Computing, Vienna, Austria) [14]. Normality of data was tested using the Shapiro–Wilk test, and checked qualitatively by means of histograms and Q–Q plots. Descriptive statistics were provided for demographic characteristics and reported as mean ± standard deviation (SD) in case of normal distribution, and as median and interquartile range (IQR) otherwise. For categorical variables, we reported frequencies and corresponding percentages.

Pearson correlation coefficient (r) was used to evaluate the linear relationship between CPET-VO_2peak_ and Questionnaire-VO_2peak_ at T_0_ [15].

To evaluate the test–retest reliability of the questionnaires, the Intraclass Correlation Coefficient (ICC) with 95% confidence interval (95%-CI) was determined (Two Way Mixed, Absolute Agreement, single measurement) [16]. The Standard Error of the Measurement (SEM, see Additional file 1: equations) [17] is a measure related to ICC, but clinically easier to interpret (expressed in the same unit as of the measurement of interest (VO_2peak_)). The ICC and SEM were calculated between T_0_ and T_1_ for all questionnaires in all patient groups together, and for each patient group separately. An ICC < 0.50 indicates poor test–retest reliability, 0.50–0.75 indicates moderate test–retest reliability, 0.75–0.90 indicates good test–retest reliability, and > 0.90 indicates excellent test–retest reliability [16]. The higher the ICC, the lower the SEM and vice versa, but there is no standard measure for the SEM as it depends on the standard deviation of the data.

In addition, Bland–Altman plots were used to present systematic errors with 95% limits of agreement (95%-LoA), by plotting the difference between Questionnaire-VO_2peak_ at T_0_ and T_1_ against the mean Questionnaire-VO_2peak_ from T_0_ and T_1_ [18].

## Results

In this study, 213 subjects participated. A total of 73 subjects did not return the T_1_-questionnaire, resulting in a response rate of 66%. 11 subjects returned it after > 31 days from T_0_ and, although we did not explicitly question, two subjects reported on paper to have changed CRF due to a COVID-19 infection and were excluded as well. As such, a total of 127 participants (84 men and 43 women) were included for analysis. The time between completing the questionnaires and CPET ranged from 11 to 31 days.

Since the data collection of some patient groups was completed sooner, we continued the data collection until a group of at least n = 26 was reached for every included patient group (pulmonary, oncology, cardiac and healthy subjects). The total study population’s age ranged from 19 to 84 years. Ancora Health included healthy subjects during the COVID-19 period, using viral filters (MicroGard II, Vyaire Medical GmbH) resulting in inaccurate data, as such we omitted VO_2peak_ data of this group [19]. As mentioned before, oncology patients were included from the outpatient clinic and did not perform CPET as part of standard care. Therefore, we present the CPET data from the total group without the healthy subjects and oncology patients. In the so-obtained population, the median VO_2peak_ was 21.94 (16.89–31.29; IQR) ml/kg/min, which is 94.1 (85.7–134.5)% of the predicted reference value for healthy Dutch persons of the same age and sex [20]. Anthropometrical data, CPET data and questionnaire data are presented in Tables 1 and 2. Data of VSAQ and DASI questionnaires can be found in Additional file 2: Table S1.

**Table 1:** Participant characteristics

Variable	Pulmonary patients	Oncology patients	Cardiac patients	Healthy subjects	Total population
*n*	32	41	28	26	127
*Anthropometrical data*					
Gender					
Male	15 (46.9%)	27 (65.9%)	21 (75.0%)	21 (80.8%)	84 (66.1%)
Female	17 (53.1%)	14 (34.1%)	7 (25.0%)	5 (19.2%)	43 (33.9%)
Age (years)	64 (53–72)	62 (51–73)	64 (57–70)	49 (38–60)	62 (49–70)
Height (cm)	170 (161–178)	175 (169–183)	175 (171–180)	179 (173–183)	175 (168–182)
Weight (kg)	79 (71–91)	81 (71–91)	80 (72–93)	73 (68–81)	79.2 (69.7–90.2)
BMI (kg*/*m)	27.6 (24.3–30.5)	26.4 (22.8–29.5)	26.1 (23.5–28.7)	23.0 (22.0–25.0)	25.3 (23.0–28.5)
COPD, GOLD classification					
None	25 (78.1%)	39 (95.1%)	27 (96.4%)	26 (100.0%)	117 (92.1%)
GOLD I	3 (9.4%)	0 (0.0%)	1 (3.6%)	0 (0.0%)	4 (3.1%)
GOLD II	3 (9.4%)	2 (4.9%)	0 (0.0%)	0 (0.0%)	5 (3.9%)
GOLD III	1 (3.1%)	0 (0.0%)	0 (0.0%)	0 (0.0%)	1 (0.8%)
GOLD IV	0 (0.0%)	0 (0.0%)	0 (0.0%)	0 (0.0%)	0 (0.0%)
Use of β-blocker					
Yes, n (%)	7 (21.9%)	4 (9.8%)	12 (42.9%)	26 (100.0%)	23 (18.1%)
No, n (%)	25 (78.1%)	37 (90.2%)	16 (57.1%)	0 (0.0%)	104 (81.9%)
*CPET data*					
FEV_1_ (L)	2.22 (1.83–3.24)	*	3.35 (2.71–3.84)	†	3.72 (2.79–4.83)‡
FVC (L)	3.10 (2.61–4.08)	*	4.14 (3.67–4.92)	†	2.95 (2.12–3.76)‡
Maximal workload (W)	106 (76–181)	*	173 (144–274)	†	143 (94–227.5)‡
VO_2peak_ (ml*/*kg*/*min)	17.47 (15.91–27.81)	*	24.92 (20.80–40.23)	†	21.94 (16.89–31.29)‡
HR_peak_ (beat*/*min)	142 (129–158)	*	153 (124–172)	†	148 (126–164) ‡
RER (VCO_2_/VO_2_)	1.08 (0.98–1.13)	*	1.12 (1.07–1.21)	†	1.10 (1.02–1.16)‡
VO_2peak_ reference^ (ml/kg/min)	26.76 (20.20–32.52)	30.25 (25.32–35.12)	32.86 (26.78–35.94)	40.16 (36.10–45.50)	32.53 (25.32–38.05)
% of the reference VO_2peak_	83.0 (66.8–107.2)	*	89.6 (73.3–117.1)	†	94.1 (85.7–134.5)‡

Results are displayed as n (%) and as median (IQR)

Missing information, number of subjects: FEV_1_, 1; FVC, 1

*cm* centimetres, *COPD* chronic obstructive pulmonary disease, *CPET* cardiopulmonary exercise testing, *FEV*_*1*_ forced expiratory volume in 1s, *FVC* forced vital capacity, *GOLD* Global initiative for chronic obstructive lung disease, *HR* heartrate, *kg* kilograms, *kg/m*^*2*^ kilograms per square meter, *L* litres, *min* minutes, *ml* millilitres, *n* number of subjects, *RER* respiratory exchange ratio, *T0* baseline measurement, *T1* second measurement, *VO*_*2peak*_ peak oxygen uptake, *W* watts

^*^Oncology patients did not perform a CPET

^†^Most subjects (unknown number) in the healthy population performed a CPET with a viral filter during the COVID-19 period, resulting in unreliable CPET/spirometry parameters. To prevent confusion, we chose to omit these variables

^‡^Oncology patients excluded. Moreover, most subjects (unknown number) in the healthy population performed a CPET with a viral filter during the COVID-19 period, resulting in unreliable CPET parameters. Given this inaccuracy, the total group for this variable is only based on pulmonary and cardiac patients

^The prediction model for VO_2peak_ of the LowLands Fitness Registry was used as a reference value [20]

**Table 2:** Intraclass correlations of the questionnaires between T_0_ and T_1_

Variable	Pulmonary patients	Oncology patients	Cardiac patients	Healthy subjects	Total population
*n*	32	41	28	26	127
FitMáx	0.96 (0.92–0.98)	0.93 (0.88–0.96)	0.98 (0.96–0.99)	0.94 (0.88–0.97)	0.97 (0.96–0.98)
VSAQ	0.90 (0.81–0.95)	0.95 (0.90–0.97)	0.89 (0.78–0.95)	0.83 (0.67–0.92)	0.94 (0.92–0.96)
DASI	0.84 (0.68–0.92)	0.87 (0.77–0.93)	0.95 (0.91–0.98)	$	0.90 (0.85–0.93) $

The ICC is presented as its output with 95%-CI

DASI; Duke Acitivity Status Index, VSAQ; Veterans Specific Activity Questionnaire

^$^As known, the DASI has a ceiling effect, resulting in the maximal score in almost all healthy subjects preventing accurate examination of the (intraclass) correlation. As such, data of the DASI for healthy subjects are omitted for analysis

The FitMáx-VO_2peak_ strongly correlated (r = 0.94 (0.91–0.97); 3.70 SEE ml/kg/min) with CPET-VO_2peak_. The correlation of the VSAQ and DASI with CPET-VO_2peak_ was lower (r = 0.85 (0.76–0.91); 5.89 SEE ml/min/kg and r = 0.76 (0.63–0.85); 6.99 SEE ml/min/kg respectively), as was expected from the results of the validation study [8].

### Test–retest reliability

**Fig. 1:**
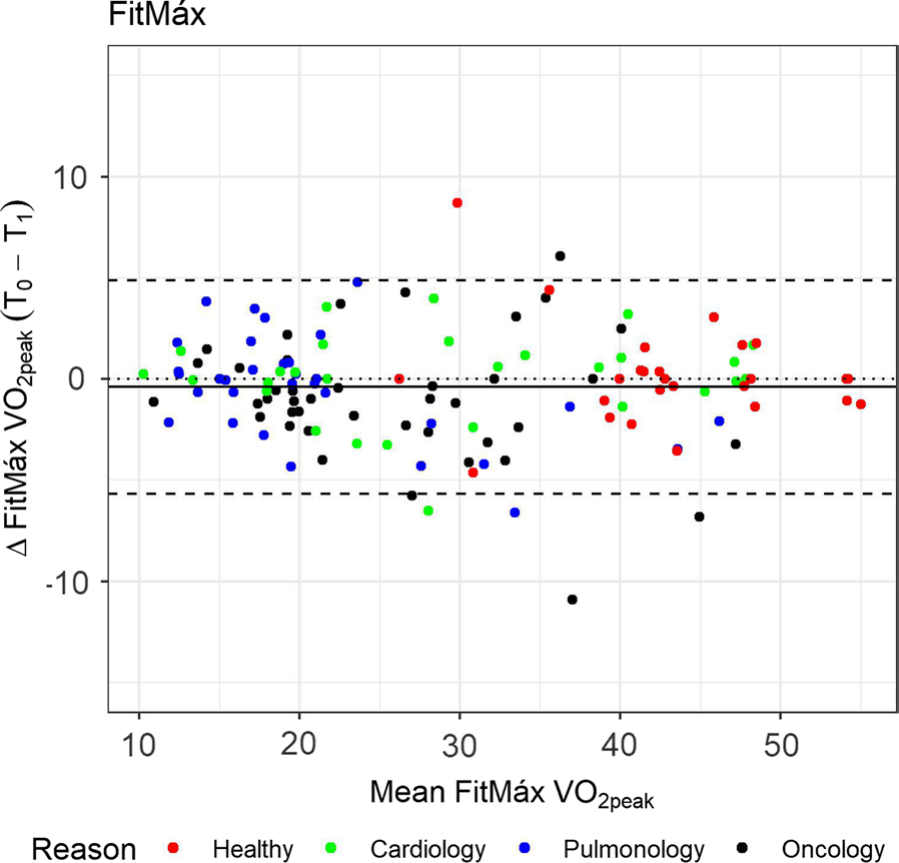
Bland–Altman plot for the FitMáx questionnaire. *Notes* The colors indicate the reason of the CPET visit. The dashed line represent the limits of agreement (− 1.96 to 1.96 SD). The solid line represents bias and the dotted line is the zero bias line

The ICC’s and corresponding 95%-CI for each patient group are displayed in Table 2. The ICC of the FitMáx-VO_2peak_ between T_0_ and T_1_ in the total population, was 0.97 (0.96–0.98). As a sensitivity analysis, we performed our ICC analysis in a two-way model examining potential systematic difference and found similar results, as expected. We found similar high ICC values in the VSAQ [0.94 (0.92–0.96)] and DASI [0.90 (0.85–0.93)] (more information in Additional file 2: Table S1). A Bland–Altman plot is provided in Fig. 1 (Additional file 3: Figure S1 for all questionnaires) showing the difference between the two values of FitMáx-VO_2peak_ at T_0_ and T_1_ against their mean. The mean difference was − 0.39 (95%-LoA − 5.68 to 4.84 ml/kg^/^min), 0.31 (95%-LoA − 8.75 to 9.37) and 0.20 (95%-LoA − 5.56 to 5.96) for FitMáx, VSAQ and DASI respectively.

## Discussion

The use of PROMs to assess CRF seems a simple, safe and cost-effective alternative for objective measurement using CPET in clinical settings [7]. The applicability of such PROMs collected via self-reported questionnaires depends upon several clinometric properties. An important aspect in the validation of a new questionnaire is the test–retest reliability. The FitMáx showed an excellent test–retest reliability between the VO_2peak_ estimated at T_0_ and T_1_, with an ICC of 0.97 (0.96–0.98; IQR) in the total population. In the different patient groups the ICC ranged from 0.93 to 0.98 for FitMáx, 0.83–0.95 for VSAQ and 0.84–0.95 for DASI. The ICC (and thus SEM) support the precision and reliability of the FitMáx and VSAQ and DASI.

A study by Ravani et al. [21] assessed the test–retest reliability of the DASI. The study was performed in pre‐dialysis patients and patients who received a kidney transplant, and obtained an ICC of 0.71 and 0.81, respectively. These ICC values were lower than the ICC value(s) we found in the current study. This difference may be caused by the 6‐month window they used in their study, which could have resulted in true CRF changes and therefore lower reliability [21].

### Strengths

The strength of the current study lies in the diverse study population. We initially included healthy subjects, oncology, pulmonary, and cardiac patients. Although oncology patients and healthy subjects did not perform (valid) CPET, a wide range of VO_2peak_ values was observed in the current study population. The VO_2peak_ ranged from (extremely) low to above average [21.94 (9.8–53.3)]. The FitMáx proves to be widely applicable in a clinical population, with both low and high VO_2peak_. Moreover, the ICC values of the FitMáx show little variance in the several subject groups. Therefore we can conclude that the ICC is independent of the CPET-VO_2peak_ and the different patient groups to estimate CRF. At last, we ensured minimized ‘subject expectancy effect’ as the participants were not told that this study aimed to determine the test–retest reliability of the FitMáx, but that they could possibly be approached a second time for the purpose of this study.

### Clinical applicability

The FitMáx is an inexpensive tool with low burden for subjects to assess CRF. Moreover, the questionnaire proves to be effective in various populations and provides information on daily life activities in several dimensions (intensity, frequency and duration). The current study shows that the FitMáx is reliable to assess CRF over time when no change in CRF has occurred. This makes FitMáx a useful tool to assess self-reported CRF among patients and healthy subjects in clinical settings.

### Limitations

The study reached a response rate of only 66%. This might be explained by the assumption of patients that they already completed the exact same questionnaires before. The test–retest period used in the current study was on average 18 days, which could have been too short to prevent subjects from remembering the response of the FitMáx from memory. However, following recommendations, we have deliberately chosen for this short recall period in order to reduce reporting error in estimates of CRF due to fluctuating experienced physical fitness, especially in patients [2, 22]. The small sample size prohibited statistical testing to compare the ICC between questionnaires. Although inspection of the ICC in supplementary material might suggest a higher reproducibility for FitMáx in most patient groups, all three questionnaire revealed high ICC values. This possible difference may not be statistically or clinically relevant.

## Conclusion

The FitMáx proves to be highly reliable in repeated measures to assess CRF of patients with different conditions and healthy subjects, when no change in CRF was expected. This increases the applicability and clinical usefulness of the FitMáx.

### Supplementary Information

**Additional file 1: **Equations used to calculate the Standard Error of the Estimate (SEE) and  the Standard Error of the Measurement (SEM).

**Additional file 2: Table S1. **Questionnaire data between T0 and T1.

**Additional file 3: Fig. S1. A–C** Bland-Altman plot for the FitMáx, VSAQ and DASI questionnaire. Notes: The colors indicate the reason of the CPET visit. The dashed line represent the limits of agreement (− 1.96 to 1.96 SD). The solid line represents bias and the dotted line is the zero bias line.

## Data Availability

The datasets used and/or analysed during the current study are available from the corresponding author on reasonable request.

## Abbreviations

BMI: Body mass index
cm: Centimetres
COPD: Chronic obstructive pulmonary disease
CPET: Cardiopulmonary exercise testing
CRF: Cardiorespiratory fitness
DASI: Duke activity status index
FEV1: Forced expiratory volume in 1s
FVC: Forced vital capacity
FitMáx: FitMáx©-questionnaire
GOLD: Global initiative for chronic obstructive lung disease
HR: Heartrate
ICC: Intraclass correlation coefficient
IQR: Interquartile range
kg: Kilograms
kg/m^2^: Kilograms per square meter
L: Liters
m: Meters
min: Minutes
ml: Milliliters
MMC: Máxima medical centre
N: Number of subjects
PROMs: Patient-reported outcome measures
r: Pearson’s correlation
RER: Respiratory exchange ratio
SD: Standard deviation
SEE: Standard error of the estimate
SEM: Standard error of the measurement
T_0_: Baseline measurement
T_1_: Second measurement
VO_2peak_: Oxygen uptake at peak exercise
VSAQ: Veterans Specific Activity Questionnaire
W: Watts
95%-CI: 95% Confidence interval
95%-LoA: 95% Limits of Agreement
