# Supplementary material for: Test–retest reliability of the FitMáx©-questionnaire in a clinical and healthy population
Source: J Patient Rep Outcomes. 2024 Jan 4;8:3. doi: 10.1186/s41687-023-00682-9 (PMC10767039; doi:10.1186/s41687-023-00682-9)
Supplement: Supplementary file 1 — Additional file 1: Equations used to calculate the Standard Error of the Estimate (SEE) and the Standard Error of the Measurement (SEM). [file 41687_2023_682_MOESM1_ESM.docx]

**Supplementary Equation**

$$\boldsymbol{SEE}=\sqrt{\frac{(1-\rho^{2})*\sum\left( Y-\mu_{Y} \right)^{2}}{N-2}}$$

$$\rho=Pearson correlation coefficient between CPET VO_{2/kg} and Questionnaire VO_{2/kg}$$

$$Y=CPET VO_{2/kg}$$

$$\mu_{Y}=Mean CPET VO_{2/kg}$$

$$N=Sample Size$$

$$\boldsymbol{SEM}=\sqrt{\frac{SS_{total}}{\left( N-1 \right)}}*\sqrt{1-ICC}$$

$$SS_{total}=s_{total}^{2}*(N-1)$$

$$N=Sample size$$

$ICC=Intraclass correlation$
