# Supplementary material for: Test–retest reliability of the FitMáx©-questionnaire in a clinical and healthy population
Source: J Patient Rep Outcomes. 2024 Jan 4;8:3. doi: 10.1186/s41687-023-00682-9 (PMC10767039; doi:10.1186/s41687-023-00682-9)
Supplement: Supplementary file 2 — Additional file 2: Table S1. Questionnaire data between T0 and T1. [file 41687_2023_682_MOESM2_ESM.docx]

Supplementary Table 1. Questionnaire data between T_0_ and T_1_

| *Variable* | *Pulmonary Patients* | *Oncology Patients* | *Cardiac Patients* | *Healthy Subjects* | *Total Population* |
| --- | --- | --- | --- | --- | --- |
| *n* | 32 | 41 | 28 | 26 | 127 |
| CPET VO_2peak_ | 17.47 (15.91-27.81) | ^ | 24.92 (20.80-40.23) | ‡ | 21.94 (16.89-31.29) † |
| FitMáx VO_2peak_ T_0_ | 19.36 (15.51-23.15) | 24.13 (19.10-30.82) | 27.20 (19.84-39.74) | 42.46 (39.68-48.03) | 26.23 (19.12-39.53) |
| FitMáx VO_2peak_ T_1_ | 19.05 (15.45-21.70) | 24.29 (19.89-32.64) | 27.75 (19.81-39.05) | 42.76 (40.43-47.82) | 27.76 (19.05-39.74) |
| VSAQ VO_2peak_ T_0_ | 15.05 (10.75-19.84) | 19.27 (14.04-24.40) | 27.00 (15.94-37.20) | 42.66 (37.05-50.65) | 21.93 (15.05-36.79) |
| VSAQ VO_2peak_ T_1_ | 15.58 (11.34-18.99) | 18.53 (14.04-24.82) | 24.57 (15.42-37.52) | 42.66 (36.50-50.80) | 21.09 (15.00-36.91) |
| DASI VO_2peak_ T_0_ | 23.45 (17.64-28.01) | 23.45 (17.32-31.40) | 28.82 (23.07-34.63) | $ | 25.54 (17.69-31.40) $ |
| DASI VO_2peak_ T_1_ | 23.02 (17.64-27.96) | 23.07 (19.76-31.40) | 33.01 (24.90-34.63) | $ | 25.38 (19.58-31.40) $ |
| ***Correlations (CPET vs FitMáx/DASI/VSAQ)*** | | | | | |
| FitMáx T_0_ r (95% CI) | 0.89 (0.78-0.94) | ^ | 0.97 (0.93-0.98) | ‡ | 0.94 (0.91-0.97) † |
| VSAQ T_0_ r (95% CI) | 0.84 (0.69-0.92) | ^ | 0.82 (0.65-0.92) | ‡ | 0.85 (0.76-0.91) † |
| DASI T_0_ r (95% CI) | 0.73 (0.51-0.86) | ^ | 0.75 (0.51-0.88) | ‡,$ | 0.76 (0.63-0.85) †,$ |
| ***SEE (CPET vs FitMáx/DASI/VSAQ)*** | | | | | |
| FitMáx | 4.18 | ^ | 3.19 | ‡ | 3.70 † |
| VSAQ | 4.96 | ^ | 7.00 | ‡ | 5.89 † |
| DASI | 6.23 | ^ | 7.87 | ‡,$ | 6.99 † |
| ***Intraclass correlations (T_0_ vs T_1_)*** | | | | | |
| FitMáx | 0.96 (0.92-0.98) | 0.93 (0.88-0.96) | 0.98 (0.96-0.99) | 0.94 (0.88-0.97) | 0.97 (0.96-0.98) |
| VSAQ | 0.90 (0.81-0.95) | 0.95 (0.90-0.97) | 0.89 (0.78-0.95) | 0.83 (0.67-0.92) | 0.94 (0.92-0.96) |
| DASI | 0.84 (0.68-0.92) | 0.87 (0.77-0.93) | 0.95 (0.91-0.98) | $ | 0.90 (0.85-0.93) $ |
| ***SEM (T_0_ vs T_1_)*** | | | | | |
| FitMáx | 1.81 | 2.27 | 1.52 | 1.75 | 1.91 |
| VSAQ | 2.95 | 2.10 | 4.17 | 3.82 | 3.25 |
| DASI | 2.53 | 2.49 | 1.44 | $ | 2.23 $ |

Notes: VO_2peak_ data is presented as the median and interquartile range (Q_1_-Q_3_). The Pearson’s correlation r and ICC are presented as its output with 95% CI*.*

Abbreviations: CPET, Cardio Pulmonary Exercise Test; DASI, Duke Activity Status Index; ICC, Intraclass Correlation Coefficient; SEM, Standard Error of Measurement; VO_2peak_, peak oxygen uptake; VSAQ, Veteran Specific Activity Questionnaire.

^ Oncology patients did not perform a CPET and were included only for the questionnaire on T_0_ and T_1_.

‡ Most subjects (unknown number) in the healthy population performed a CPET with a viral filter during the COVID-19 period, resulting in unreliable CPET/spirometry parameters. To prevent confusion, we chose to omit these variables.

† Most subjects (unknown number) in the healthy population performed a CPET with a viral filter during the COVID-19 period, resulting in unreliable CPET parameters. Given this inaccuracy, and given that oncology patients did not perform a CPET, the total group (and its correlation with the CPET) is only based on pulmonary and cardiac patients.

$ As known, the DASI has a ceiling effect, resulting in the maximal score in almost all healthy subjects preventing accurate examination of the (intraclass) correlation. As such, data of the DASI for healthy subjects are omitted for analysis.
