# Supplementary figures and images for: Test–retest reliability of the FitMáx©-questionnaire in a clinical and healthy population
Source: J Patient Rep Outcomes. 2024 Jan 4;8:3. doi: 10.1186/s41687-023-00682-9 (PMC10767039; doi:10.1186/s41687-023-00682-9)

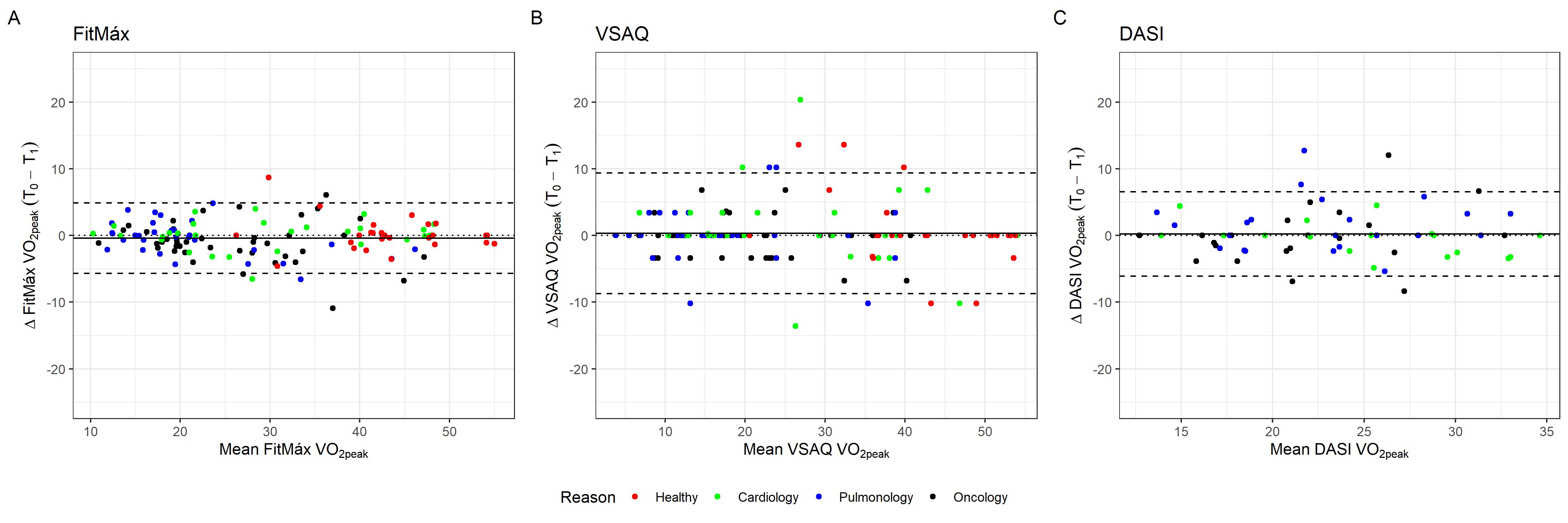

Supplement: Supplementary file 3 — Additional file 3: Fig. S1. A–C Bland-Altman plot for the FitMáx, VSAQ and DASI questionnaire. Notes: The colors indicate the reason of the CPET visit. The dashed line represent the limits of agreement (− 1.96 to 1.96 SD). The solid line represents bias and the dotted line is the zero bias line. [file 41687_2023_682_MOESM3_ESM.jpg]
